# Pediatric kidney transplantation using donors after circulatory death: a national experience from Spain

**DOI:** 10.1007/s00467-026-07235-4

**Published:** 2026-04-14

**Authors:** María Herrero-Goñi, Mireia Aguirre Meñica, Alejandro Zarauza Santoveña, Victor Perez-Beltran, Yolanda Calzada, David Canalejo González, Ana Adell Sales, Olalla Alvarez Blanco, Iñaki Bilbao-Villasante, Spanish Pediatric Registry of Renal Replacement Treatment (REPIR I), Gema Ariceta, Carlota Fernández Clambor, Marta Gil, Mercedes López González, María Luisa Matoses Ruiperez, Francisco Antonio Nieto Vega, Flor Angel Ordóñez, Adela Rodríguez Barba, Francisco Vela Enríquez, Carmen Vicente Calderón

**Affiliations:** 1 Department of Pediatric Nephrology, IIS BioBizkaia Health Research Institute, Cruces University Hospital, University of Deusto, University of the Basque Country UPV/EHU, Cruces Place, 48903 Barakaldo, Bizkaia Spain; 2 Department of Pediatric Nephrology, IIS BioBizkaia Health Research Institute, Cruces University Hospital, Barakaldo, Bizkaia Spain; 3 Department of Pediatric Nephrology, La Paz University Hospital, Madrid, Spain; 4 Department of Pediatric Nephrology, University Hospital Vall d’Hebron, Barcelona, Spain; 5 Department of Pediatric Nephrology, Sant Joan de Déu Hospital, Barcelona, Spain; 6 Department of Pediatric Nephrology, Virgen del Rocío University Hospital, Sevilla, Spain; 7 Department of Pediatric Nephrology, La Fe University and Polytechnic Hospital, Valencia, Spain; 8 Department of Pediatric Nephrology, Gregorio Marañón Hospital, Madrid, Spain; 9 Department of Anesthesiology and Intensive Care, Department of Transplantation, Regional Transplant Coordination, Cruces University Hospital, Barakaldo, Bizkaia Spain

**Keywords:** Kidney transplant, Donation after circulatory death, Donation after brain death, Pediatrics, Delayed graft function

## Abstract

**Background:**

The shortage of pediatric kidney donors has increased interest in donation after circulatory death (DCD) as an alternative for transplantation.

**Methods:**

This multicenter, retrospective study analyzed all pediatric kidney transplants (KT) from DCD donors performed in Spain between 2013 and 2024 in recipients under 18 years and compared them with 490 KTs from donation after brain death (DBD) donors during the same period.

**Results:**

Sixty-four DCD KTs were included. Delayed graft function (DGF) occurred in 12% of cases. DGF risk was higher with donor age > 40 years (*p* > 0.05) and in grafts retrieved using the rapid recovery (RR) extraction technique (*p* < 0.05). The median functional warm ischemia time was 13 min (IQR:9–18) and was not associated with an increased risk of DGF. The median cold ischemia time (CIT) was 12.8 h (IQR:9.4–17), and longer CIT was associated with a nonsignificant increase in DGF risk. No DGF occurred when CIT was < 14 h and no additional risk factors were present. Recipients with DGF had a lower estimated glomerular filtration rate one month post-transplant (*p* < 0.05), but no significant difference at one year. Five-year graft survival rates were not significantly lower in DCD compared to DBD KTs (89.7% vs*.* 88.5%).

**Conclusions:**

Although DGF risk was associated with older donor age, RR technique, and longer ischemia times, it did not affect one-year graft function. Graft survival with DCD donors did not appear inferior to that with DBD. DCD KT appears feasible in pediatric recipients and may help expand donor availability under carefully selected conditions.

**Graphical abstract:**

**Figure:**
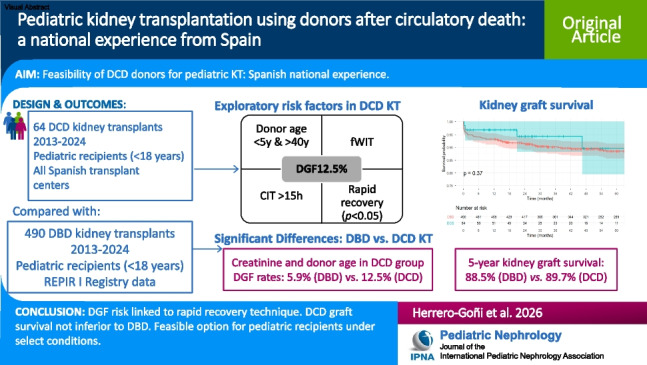
A higher resolution version of the Graphical abstract is available as Supplementary information

**Supplementary Information:**

The online version contains supplementary material available at 10.1007/s00467-026-07235-4.

## Introduction

Kidney transplantation (KT) is the best treatment for children with kidney failure and should be considered the first-line therapy [1–4]. This approach not only improves patient survival [4–6], but also enhances neurocognitive development, growth, and quality of life, in addition to offering economic benefits [7–9].

In recent years, the number of donors has declined; according to Spanish National Transplant Organization (ONT) figures in their 2024 report, KTs from donors after brain death (DBD) decreased from 2057 annually in 2015 to 1785 in 2024 (www.ont.es). This situation has been accompanied by an increasing number of complex pediatric recipients, making pediatric transplantation more challenging. Although Spain has a policy prioritizing transplantation for pediatric patients under 18 years of age, a gap remains between the demand and the availability of deceased donor grafts. This situation has prompted the development of various strategies to expand the donor pool.

In some countries, donation after circulatory death (DCD) has emerged as a growing practice to alleviate organ shortages and the imbalance between donors and recipients [10–12]. In Spain, the DCD program began in 2012. This marked the culmination of efforts that led to the publication of a national consensus document on DCD use by the Spanish ONT, (www.ont.es), aimed at regulating DCD practice, alongside the establishment of the legal framework in Royal Decree 1723/2012 (https://www.boe.es/eli/es/rd/2012/12/28/1723/con), which governs organ procurement, clinical use, and territorial coordination, while defining quality and safety requirements. The decree (Online Resource 1) clearly outlines the conditions for declaring death by cardiocirculatory criteria (previously regulated in Law 30/1979 of October 27 on organ retrieval and transplantation). Since then, utilization of DCD donors has steadily increased, accounting for 35% of all donations by 2023. In adults, long-term patient and graft survival rates following transplantation with DCD kidneys have been reported to be generally similar to those observed with grafts from DBD, despite higher rates of delayed graft function (DGF) and primary non-function [13].

Encouraged by the positive outcomes observed in adults, the use of DCD donors has begun to be explored in pediatric transplantation. However, in contrast to the extensive literature available for adults, evidence in the pediatric population remains limited. Therefore, we analyzed all pediatric KTs from DCD donors performed in Spain. The aim of this study is to describe the characteristics and outcomes of pediatric KT recipients from DCD donors and to compare them with 490 KTs from DBD donors during the same period, as part of a feasibility assessment of DCD use in the pediatric setting.

## Materials and methods

### Study design

This is a retrospective, multicenter, descriptive study that includes all pediatric patients (≤ 18 years of age) who received a KT from a DCD donor between January 2013 and December 2024 across all national pediatric KT programs in Spain. Data from patients receiving DBD kidneys in the same period were extracted from the Spanish Pediatric Registry of Renal Replacement Treatment, REPIR I (www.aenp.es/registros).

### Clinical data

Data were collected through a review of electronic patient medical records. Recipient characteristics included demographics, age, height, weight, primary kidney disease, dialysis modality, duration of dialysis, waiting time on the transplant list, and immunosuppressive treatment. Graft outcome parameters were collected for up to 10 years post-transplant, including graft function as estimated glomerular filtration rate (eGFR), calculated using the bedside IDMS-Traceable Schwartz equation [14], number of transplants, and graft loss and cause, if applicable.

Donor data included demographics, cause of death, and pre-mortem serum creatinine. Additional variables collected included human leukocyte antigen (HLA) mismatches, cold and warm ischemia times (WIT), rapid recovery (RR) or normothermic (NT) extraction technique and type of donation (multiorgan or single-organ donation).

### Definitions

Total Warm Ischemia Time (tWIT): Defined as the time from initiation of withdrawal of life-sustaining measures to initiation of organ preservation maneuvers [12].

Functional warm ischemia time (fWIT): Defined as the interval from the onset of significant hypoperfusion to the initiation of preservation procedures [12, 15].

Cold ischemia time (CIT): Defined as the time from cross-clamp to reperfusion after organ implantation in the recipient [16].

Delayed Graft Function (DGF): Defined as the requirement for at least one dialysis session during the first week after KT [16].

### Statistical analysis

Continuous variables were expressed as mean ± standard deviation (SD) if normally distributed, and as median and interquartile range [IQR: 25th–75th] if not normally distributed. To determine normality, the Shapiro–Wilk test was applied. For comparisons, t-test or Wilcoxon-Mann–Whitney test was used depending on whether the variable followed a normal distribution or not. Kaplan–Meier survival curves were constructed to estimate kidney allograft and patient survival. A p-value of < 0.05 was considered statistically significant and statistical analyses were performed using R version 4.4.0 (R Core Team, 2025). All patient-specific information was de-identified to ensure confidentiality.

### Consent

All patients were informed about this study. Written informed consent was obtained from parents or legal guardians and assent was obtained from children over 12 years of age for the publication of this research. There were no refusals. Additionally, this retrospective study involving human participants was approved by the Ethics Committee for Clinical Research of Euskadi (ID: PI2024227).

## Results

In Spain, 554 deceased-donor pediatric KTs were performed across 7 pediatric KT programs between 2013 and 2024 (data obtained from REPIR I). Of these, 64 patients received kidneys from 58 DCD donors, representing 11.5% of all deceased-donor pediatric KTs. All of these patients were alive at the end of the study period.

### Recipients’ characteristics (Table 1, Online Resource 1)

Table 1 summarizes the characteristics of 64 pediatric recipients who received a KT from 58 DCD donors. The median follow-up time after transplantation was 26.5 months (IQR: 15.9–53.8). The median age at transplantation was 10.7 years (IQR: 4.7–14.2). The median time of dialysis prior to KT was 12.4 months (IQR: 5.3–25.4). The median waiting time on the transplant list was 3 months (IQR: 1–8).

**Table 1:** Clinical characteristics of 64 recipients of KTs from 58 DCD donors

Recipients´ characteristics	Median [IQR: 25th, 75th] or number (%)
**Age at transplantation (year)**	10.7 [4.7;14.2]
**Sex (Male/Female)**	42 (65.6%)/22 (34.4%)
**Ethnicity**	
White	44 (68.75%)
Arabic	11 (17.2%)
Hispanic	6 (9.4%)
Other	3 (4.7%)
**Cause of kidney disease**	
Kidney hypoplasia/dysplasia/CAKUT	21 (32.8%)
Focal segmental glomerulosclerosis (FSGS)	7 (10.9%)
Other Glomerular disease	14 (21.8%)
Nephronophthisis	8 (12.5%)
Ischemia/thrombosis	4 (6.25%)
Congenital nephrotic syndrome	4 (6.25%)
Polycystic kidney disease	3 (4.7%)
Other	11 (17.2%)
**Weight (kilogram)**	30 [15.7;46.25]
≤ 10	2
10.1–15	12
> 15.1	50
**Height (cm)**	132.5 [100.25;157.6]
**Number of Kidney transplant**	
First	58 (90.6%)
Second	5 (7.8%)
Third or more	1 (1.6%)
**Kidney replacement therapy**	
Preemptive transplant	13 (20.3%)
Peritoneal dialysis	19 (29.7%)
Hemodialysis	32 (50%)
**Time on dialysis (peritoneal dialysis or hemodialysis) (months)**	12.4 [5.3;25.4]
**Time on the waiting list (months)**	3 [1;8]
Preemptive transplant	1.5 [0.9;4.4]
Peritoneal dialysis	7 [3;15.1]
Hemodialysis	2.1 [0.95;6.3]
Hypersensitized (cPRA ≥ 98%)	1 (1.56%)
**HLA mismatches**	
A mismatches (0,1,2)	3, 29, 32 (4.7%, 45.3%, 50%)
B mismatches (0,1,2)	0, 20, 44 (0%, 31.2%, 68.7%)
DR mismatches (0,1,2)	12, 31, 21 (18.7%, 48.4%, 32.8%)

*KT* Kidney transplant, *DCD* Donation after circulatory death, *N* Number, *CAKUT* Congenital anomalies of the kidney and urinary tract, *FSGS* Focal and segmental glomerulosclerosis, *cPRA* Calculated panel reactive antibody, *HLA* Human leukocyte antigen

All patients received standard induction immunosuppression, with basiliximab used in most cases (72%). Fourteen patients (21.9%) received anti-thymocyte globulin according to center-specific protocols, primarily for repeated KT (6 cases), focal segmental glomerulosclerosis (1 case) or based on center preference. The initial immunosuppression schedule included calcineurin inhibitors in 62 patients (96.8%, tacrolimus in 61) and mycophenolate mofetil or mycophenolic acid in 62 cases (96.8%). All patients received corticosteroids according to the protocol of each center. Additionally, 5 patients received plasmapheresis/immunoadsorption, immunoglobulins, rituximab or eculizumab due to the risk of baseline disease recurrence, following center-specific protocols.

### Donors’ characteristics (Tables 2, 4 and 5)

Table 2 summarizes the characteristics of 58 DCD and 400 DBD donors. The median donor age was 27.7 years (IQR: 14–45) for DCD donors vs. 21.1 years (IQR: 10.8–38.5) for DBD donors, due to a higher proportion of donors aged ≤ 5 years in the DBD group. In the DCD cohort, 15 donors (25.8%) were older than 40 years (oldest: 58 years), while 22 (37.8%) were pediatric donors (< 18 years) and 3 (5.1%) were aged < 5 years (youngest: 3 years). Mean serum creatinine levels were normal in both groups but significantly lower in the DCD group (*p* < 0.05). No differences were observed between DCD and DBD donor groups in cardiovascular risk factors (weight, BMI, diabetes mellitus, hypertension, or proteinuria). As explained in the Introduction, all DCD donors complied with current Spanish legal and ethical regulations.

**Table 2:** Characteristics of DCD and DBD donors

Donor characteristics (*n* = 64)	Donation after circulatory death Median [IQR: 25th, 75th] or number (%) *N* = 58	Donation after brain death Median [IQR: 25th, 75th] or number (%) *N* = 400	*P* value
**Age (years)**	27.7 [14;45]	21.1 [10.8;38.5]	*P* < 0.05
≤ 5 years	3 (5.1%)	52 (13.0%)	*n.s*
> 5—≤ 18 years	19 (32%)	128 (32.0%)	*n.s*
> 18—≤ 40 years	21 (36.2%)	132 (33.0%)	*n.s*
> 40 years	15 (25.8%)	88 (22.0%)	*n.s*
**Gender**			
Male	36 (62.1%)	225 (56.2%)	*n.s*
Female	22 (37.1%)	175 (43.8%)	
**Serum creatinine (mg/dL)**	0.47 [0.35;0.6]	0.7 [0.5;0.9]	*P* < 0.05
**Weight (kilogram)**	60 [34; 80]	60.0 [40.2;75.0]	*n.s*
**BMI** (kg/m^2^)	22.9 [20.7;26.0]	22.2 [18.7;25.7]	*n.s*
**Diabetes Mellitus**	2 (3.4%)	7 (1.8%)	*n.s*
**Hypertension**	3 (5.1%)	21 (5.2%)	*n.s*
**Proteinuria**	5.0 (0.0–30.0)	2.0 (0.0–24.0)	*n.s*
**Cause of death**			
Anoxia	15 (25.9%)	99 (24.8%)	*n.s*
Cerebrovascular accident	14 (24.1%)	132 (33.0%)	*n.s*
Cranioencephalic trauma	13 (22.4%)	142 (35.6%)	*P* < 0.05
Demyelinating disease	7 (12.1%)	0	*P* < 0.05
Cerebral tumor	2 (3.4%)	3 (0.8%)	*n.s*
Other	7 (12.1%)	24 (6.1%)	*n.s*
**Multiorgan donation**	56 (87.5%)	371 (92.8%)	*n.s*

*DCD* Donation after circulatory death, *DBD* Donation after brain death, *BMI* Body mass index, *N.S.* non-significant (*P* > 0.05)

### Transplantation perioperative features using grafts from DCD and DBD donors (Table 3, Online Resource 2 and 3)

In KTs from DCD and DBD donors, approximately 90% were multiorgan procurements. Median CIT was 12.8 h (IQR: 9.4–17) in DCD donors and 14 h (IQR: 12–16.5) in DBD donors, with no significant differences between groups. Comparing DCD and DBD donors, DGF rates were significantly higher in the DCD group (*p* < 0.05).

**Table 3:** Transplantation perioperative features using grafts from DCD and DBD donors

Transplantation perioperative features	Donation after circulatory death Median [IQR: 25th, 75th] or number (%) *N* = 64	Donation after brain death Median [IQR: 25th, 75th] or number (%) *N* = 490	*P* value
**CIT** (h)	12.8 [9.4;17]	14 [12;16.5]	*n.s*
**fWIT **(min)	13.06 [9;18]	N/A	N/A
**DGF**	8 (12.5%)	23/384 (5.9%)	*P* 0.03
**Transplant loss in the first month**	2 (3.1%)	21 (4.2%)	*n.s*
**Transplant loss in the first 5 years**	4 (6.2%)	53 (10.8%)	*n.s*
**Modality of organ extraction**			
Rapid-recovery	4 (6.25%)	N/A	N/A
Normothermic	60 (93.75%)		
**DGF**	8 (12.5%) *p* > 0.05		
Donors ≤ 18 years	3/27 (11.1%)	Not available	Not available
Donors > 18—≤ 40 years	1/22 (4.5%)		
Donors > 40 years	4/15 (26.7%)		
Rapid recovery extraction	2 out of 4	N/A	N/A
Normothermic extraction	6 out of 60		

*CIT* cold ischemia time, *fWIT* functional warm ischemia time, *DGF* delayed graft function, *N.S.* non-significant (*P* > 0.05), *N/A* not applicable

Regarding KTs from DCD donors, most organ procurements were performed using the normothermic technique (93.7%). Rapid recovery (RR) extraction was used in only 4 cases (6.25%) and was associated with an increased risk of DGF: 50% of patients who underwent RR extraction developed DGF, compared to 10% of those who underwent normothermic extraction (*p* < 0.019).

In KTs from DCD donors, median fWIT was 13 min (IQR: 9–18), with no significant association between fWIT and the risk of DGF (*p* = 0.9). In contrast, patients who developed DGF tended to have longer CIT, although this difference did not reach statistical significance (*p* = 0.1). Specifically, DGF was observed in 19.2% of patients with CIT ≥ 15 h, compared to 5.2% of those with CIT < 15 h (*p* < 0.05) (Online Resource 2). Patients who developed DGF had significantly lower eGFR at 1 month post-transplantation (*p* < 0.05); however, the gap between groups decreased over time, yet eGFR values tended to remain lower in the DGF group at 1 year. Additionally, no correlation was found between eGFR at 1 month or at 1 year post-transplantation and either CIT or fWIT in our series (Online Resource 3).

### Characteristics of patients with DGF (Table 4) and graft loss (Table 5) from DCD donors

In our series, 2 patients (3.1%) experienced early graft loss. One case was due to immediate arterial thrombosis in a recipient with standard thrombosis risk (case 6). The other was attributed to early T cell-mediated rejection despite standard immunosuppression with basiliximab, corticosteroids, an antimetabolite, tacrolimus, and pre-transplant prophylactic rituximab due to high recurrence risk. Anti-thymocyte globulin was initiated 3 days post-transplantation, but nephrectomy was required on day 8 due to bleeding (case 5). No cases of primary non-function were observed.

**Table 4:** Characteristics of 8 patients with DGF from DCD donors

Case	Recipient characteristics			Donor characteristics Sex/Age (years) Serum creatinine (mg/dL)	Cold (h)/ Warm ischemia time (min)	Extraction modality/Multiorgan donation	HLA mismatches: A/B/DR	Early Kidney graft loss	eGFR at 1 year (ml/min/1.73 m^2^)	eGFR at the last follow-up visit (time after transplantation) (ml/min/1.73 m^2^)	Comments
*n*	Sex/age (years)/ethnic	pre-KT treatment/ time on the waiting list (months)	Cause of ESKD								
1	F/10/ Hispanic	Preemptive/ 0.01	Unknown	F/48/0.7	16.4/14	NT/Yes	1/2/1	No	53	36.2 (4y 7 m)	
2	M/14/White	HD/2	Congenital NS	M/45/0.55	14/3	RR/No	2/1/0	No	52	53.1 (1y 7 m)	4th KT
3	M/9/White	HD/3	CAKUT	M/14/0.29	19/11	NT/Yes	1/1/2	No	104	100 (1y 5 m)	
4	M/16/Arabic	HD/1	TTP	M/49/0.6	15/12	RR/Yes	2/2/1	No	43	38 (7y)	Pyelonephritis, BK Virus Nephropathy, Thrombotic microangiopathy
5	F/16/White	Preemptive/ 0.8	FSGS	M/48/0.7	14/10	NT/Yes	2/2/1	Yes	-	-	TCMR
6	M/17/White	DP/18	TIN	M/9/0.22	21/13	NT/Yes	2/2/2	Yes	-	-	Arterial thrombosis
7	M/16/White	HD/16	Glomerular	M/32/1.7	18/13	NT/Yes	1/1/1	No	118.7	-	eGFR 109 (3y 6 m) Graft loss at 3y 10m: mixed rejection due to nonadherence
8	M/13/Arabic	HD/11	Nephronophthisis	F/15/0.4	6/28	NT/Yes	1/2/1	No	54	55 (1 year 1 month)	

*DGF* Delayed graft function, *eGFR* Estimated glomerular filtration rate, *KT* Kidney transplantation, *F* Female, *M* Male, *NS* Nephrotic syndrome, *NT* Normothermic, *RR* Rapid-recovery, *CAKUT* Congenital anomalies of the kidneys and urinary tract, *TTP* Thrombotic thrombocytopenic purpura, *HD* Hemodialysis, *PD* Peritoneal dialysis, *FSGS* Focal and segmental glomerulosclerosis, *TIN* Tubulointerstitial nephritis, *TCMR* T cell-mediated rejection, *ABMR* Antibody-mediated rejection, *Y* Year, *M* Month

**Table 5:** Characteristics of patients who lost kidney grafts from DCD donors

Case	Recipient characteristic			Donor characteristics: Sex/Age (years) Serum creatinine (mg/dL)	Cold (h)/ Warm ischemia time (min)	Extraction modality/Multiorgan donation	HLA mismatch: A/B/DR	DGF	Time after transplantation	Comments
	Sex/age (years)/ethnic	pre-KT treatment/time on waiting list (months)	Cause of ESKD							
5	F/16/White	Preemptive	FSGS	M/48/0.7	14/10	NT/Yes	2/2/1	Yes	8 days	Early kidney loss due to TCMR
6	M/17/White	PD/18	TIN	M/9/0.22	21/13	NT/Yes	2/2/2	Yes	Immediately	Early kidney loss due to arterial thrombosis
7	M/16/White	HD/16	Glomerular	M/32/1.7	18/13	NT/Yes	1/1/1	Yes	3 years 10 months	Mixed rejection due to nonadherence
9	M/13/Arabic	HD/0.5	FSGS	F/39/0.28	4.7/2	NT/Yes	2/2/2	No	2 years 6 months	TCMR and disease recurrence

*F* Female, *M* Male, *NS* Nephrotic syndrome, *NT* Normothermic, *HD* Hemodialysis, *PD* Peritoneal dialysis, *FSGS* Focal and segmental glomerulosclerosis, *TIN* Tubulointerstitial nephritis, *DGF* Delayed graft function, *TCMR* T cell-mediated rejection

Two additional patients lost their grafts at 2 and 3 years post-transplantation: one due to rejection and disease recurrence, and the other one due to rejection associated with non-adherence (cases 9 and 7, respectively). Nineteen recipients of KTs from DCD donors underwent biopsy (29.6%). Of these, only one was a protocol biopsy without kidney dysfunction. The remaining 18 were performed due to kidney dysfunction and/or BKV infection. Diagnoses included 6 rejections, 4 BK nephropathy, 3 interstitial fibrosis and tubular atrophy, and 2 acute tubular injuries. Of the rejections, 3 resulted in KT loss (Table 5); the remaining 3 comprised 1 ABMR and 2 TCMR with favorable outcomes.

**Fig. 1:**
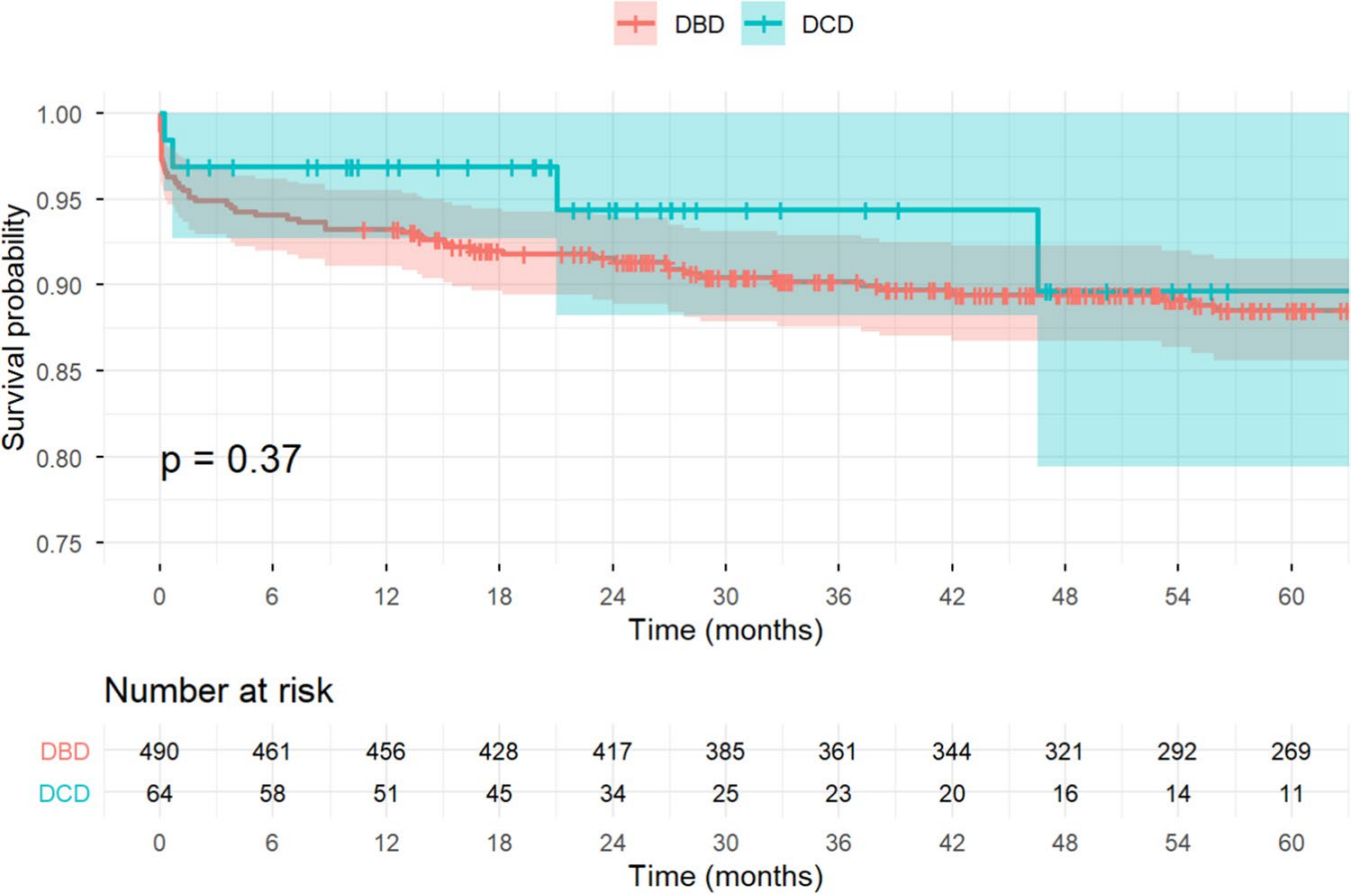
Kidney graft survival from DCD and DBD donors. DCD, donation after circulatory death; DBD, donation after brain death

Figure 1 illustrates kidney graft survival in pediatric recipients who received kidneys from DCD and DBD donors. At 3 years post-transplantation, graft survival was 94.4% in recipients of kidneys from DCD donors (*n* = 23 at risk) and 90.2% in those from DBD donors (*n* = 361 at risk). At 5 years of follow-up, graft survival remained high in both groups, with rates of 89.7% in the DCD group (*n* = 11 at risk) and 88.5% in the DBD group (*n* = 269 at risk). No significant differences in graft survival were observed between groups at either time point.

## Discussion

Although accumulating evidence supports favorable outcomes in adult KT using organs from DCD, the use of DCD kidneys in pediatric patients remains limited, primarily due to concerns regarding potentially inferior graft function [10, 17]. Considering previous reports showing that DCD donors may be associated with less favorable outcomes than DBD donors, in this study we present our national experience with 64 pediatric recipients who received KTs from DCD donors and report short-term favorable transplantation outcomes.

Previous reports in adults with kidney failure have shown that remaining on dialysis is associated with poorer patient survival compared to receiving a KT from a DCD donor [18]. In our series, 80% of patients who received a DCD kidney were on dialysis prior to transplantation (Table 1). As expected, this proportion was higher than the 60% of patients who received a DBD kidney during the same period, according to data from REPIR I.

Historically, the acceptance of DCD kidneys has been limited due to the higher risk of DGF [10, 19–23]. As expected, in our series the rate of DGF in KTs from DBD donors was significantly lower compared with DCD donors (5.9% vs. 12.5%). Based on the data presented in Tables 2 and 3, comparing donor characteristics and immediate perioperative outcomes, we found no differences in sex distribution, cardiovascular risk factors, or multiorgan donation between DBD and DCD donors. However, DBD donors were younger than DCD donors. This difference may be explained by the higher acceptance of kidneys from donors younger than 5 years in the DBD group but not in the DCD group (13% vs. 5%, respectively), due to the known surgical risk and kidney immaturity associated with very young donors [24–26].

Interestingly, donor serum creatinine levels were lower in DCD compared with DBD donors, likely reflecting the more stringent selection criteria applied when accepting DCD organs.

When comparing our series of DCD donors with two large cohorts published from the UK and USA, we found that our incidence of DGF was significantly lower (12.5%, Table 3) than in those cohorts, which reported DGF rates of 25% and 19.7%, respectively [16, 17].

We analyzed several factors that could account for this lower DGF rate in our cohort, including recipient weight, donor age, type of organ retrieval, and both cold and fWIT. Pre-mortem donor serum creatinine was excluded as a risk factor for DGF, as only donors with normal kidney function are accepted in our community (Tables 1, 2 and 3). Beyond the single significant association observed with rapid recovery, all other findings should be considered exploratory.

Interestingly, in our series, 14 recipients (21.8%) weighed less than 15 kg, including 2 who weighed less than 10 kg (Table 1). Although it is well established that small recipient size increases the risk of surgical complications and graft loss [24], we did not observe any size-related complications, DGF, or rejection episodes in this subset of patients. Therefore, our findings suggest that the use of DCD kidneys may be considered even in low-weight recipients when a more suitable donor is not available.

Regarding donor age in DCD KTs, the median donor age in our series was 27.7 years, compared with 21.7 years in the cohort reported by MacConmara and 18 years in the group described by Marlais [16, 17]. Among the youngest donors, 3 were aged 5 years or younger (Table 2). As we previously noted, although younger donors may not be ideal, no adverse outcomes were observed in this subgroup of recipients, including primary non-function or DGF [25, 26].

Conversely, 15 donors (25.8%) in our cohort were older than 40 years. While recipients of kidneys from donors over 40 years had a higher incidence of DGF, no significant differences were observed across age subgroups (Table 3). This may reflect the fact that all older donors had serum creatinine levels comparable to those of younger donors, potentially minimizing age-related risk, as reported in other series [27, 28].

In summary, despite accepting donors at both extremes of age—aged 5 years or younger and older than 40 years—no poorer outcomes attributable to donor age were observed in our series. Nevertheless, kidneys from donors aged 5–40 years may represent the optimal choice to further minimize risks associated with DCD donation.

According to the literature, NT has demonstrated better outcomes than RR [29], a technique that is now virtually obsolete in our country [15]. Our series did not record whether NT regional perfusion or NT machine perfusion was performed. However, this does not represent a significant limitation, as no clear advantage of one technique over the other has been established to date [20]. As expected, 50% of patients who underwent RR extraction developed DGF compared to 10% with NT extraction (*p* < 0.05) (Table 3). These findings support avoiding RR extraction to optimize immediate kidney function [12, 15].

Concerning ischemia time as a risk factor for DGF, the median fWIT in our cohort was 13 min, ranging from 2 to 30 min. This value is substantially lower than those reported in previous series: 17 out of 21 cases in the UK cohort had a fWIT of 30 min or more, and the mean fWIT in the US cohort was 16 min [16, 17]. As expected, DGF was more frequent among patients with longer fWIT (Online Resource 3), although the difference was not statistically significant, likely due to the short ischemia times across nearly all patients. The difference from larger published cohorts likely reflects the adoption of more recent organ retrieval and preservation techniques that reduce DGF risk. These findings underscore the importance of minimizing fWIT to reduce DGF risk and optimize outcomes.

A longer CIT has been reported to be associated with an increased risk of DGF [30]. In our cohort, we did not observe significant differences in CIT between the DCD and DBD groups. Nevertheless, within the DCD donors, the association between DGF and prolonged CIT was more apparent when CIT was ≥ 15 h (Online Resource 3). Notably, only one patient who developed DGF had a CIT of < 14 h; in this instance, the adverse outcome might be related to a fWIT of 28 min (Table 4, Case 8). These observations are in line with those reported by the MacConmara group, who proposed that maintaining CIT below 16 h could contribute to improved graft outcomes [17] (Table 5).

Based on the findings described above, the concurrence of more than one of the following risk factors—donors younger than 5 years or older than 40 years, RR extraction modality, CIT greater than 15 h, or prolonged fWIT—may have contributed to the increased likelihood of DGF. As shown in Table 4, among the 8 patients who developed DGF, 4 donors were older than 45 years (cases 1, 2, 4, and 5); kidney recovery was performed using the RR technique in 2 recipients, and CIT was ≥ 15 h in 5 cases. In one of these, the fWIT was 28 min, as previously noted (case 8). Overall, these observations suggest that DCD donors could represent a feasible option for pediatric recipients, if donor selection accounts for these potential risk factors for DGF.

Patients who developed DGF showed lower eGFR at one month post-transplantation compared to those without DGF. However, no statistically significant differences were observed at one year (Online Resource 3), consistent with prior reports [21–23]. While minimizing DGF remains crucial for early graft function, our cohort suggests no deleterious short-term impact on kidney function at one year—though these findings should be interpreted cautiously given the limited follow-up duration.

Although KT from DCD donors exhibited higher rates of DGF compared to DBD donors, no increased graft losses were observed during the first month or over the initial 5 years of follow-up in the DCD group (Table 3). In our cohort, among DCD donors, 2 patients (3.1%) experienced early graft loss—a rate lower than previously reported [17]. Two additional graft losses occurred years later and appear unrelated to DCD donor use. During follow-up, biopsy data were available for 19 patients (29.6%) in our cohort, with only 1 performed per protocol. Among those biopsied, 3 experienced unfavorable outcomes due to rejection leading to graft loss—rates comparable to the DBD group.

Overall, graft survival rates with DCD kidneys appeared like those achieved with DBD kidneys. REPIR I data reported 90.2% and 88.5% graft survival for DBD kidneys at 3 and 5 years, respectively, whereas our series showed 94.4% and 89.7% graft survival for DCD kidneys at the same time points. No significant differences were observed between the groups (Fig. 1). However, the number of cases in the DCD group was markedly lower than in the DBD group, and the groups were not matched. Therefore, these findings should be interpreted with caution. These results suggest that the use of DCD grafts may be a feasible option for pediatric patients with kidney failure, particularly in settings where access to living donation or standard DBD grafts is limited.

Moreover, graft survival from DCD donors in our series exceeded rates reported by MacConmara [17] and Marlais [16] at both 1- and 3-year follow-up (Fig. 1). This improvement may reflect advances achieved accepting DCD kidneys in recent years [5, 17]. The factors discussed above likely contributed to these enhanced outcomes.

This study has several limitations. First, the retrospective design and relatively small sample size may limit the strength of the conclusions. Although all pediatric KTs from DCD donors performed in Spain between 2013 and 2024 were included—enhancing data consistency through electronic records— protocol biopsies were unavailable to detect subclinical rejection events that might influence long-term kidney function.

Although graft survival rates in the DCD group were favorable, eGFR trajectories were not systematically evaluated. In addition, the comparison groups were not matched, which may limit the interpretation of the results. Moreover, the relatively short follow-up period warrants cautious interpretation. Larger prospective studies with extended follow-up, including protocol biopsies and eGFR assessment with matched groups, would help confirm the potential benefits of DCD donors in pediatric KT.

A shortage of potential donors for pediatric recipients is well recognized, underscoring the need to expand the donor pool while ensuring adequate graft function. The use of kidneys from DCD donors in pediatric patients remains uncommon [10, 17]. However, as observed in our series, meticulous donor selection appears to yield favorable outcomes. Expanding the use of DCD donors may help reduce waiting times for transplantation, as the broader donor pool could increase the number of available grafts, potentially enabling more KTs and improving patient survival [10]. Nonetheless, the use of DCD donors should be considered primarily in carefully selected cases or specific clinical circumstances, given the still limited pediatric experience to date [31].

In addition, to our knowledge, this is the largest study of DCD donors and pediatric recipients using data from Spain to date, showing graft survival rates that appear similar to those observed with DBD kidney transplantation.

This study contributes to understanding the use of DCD donors and suggests favorable short-term outcomes with DCD kidneys in pediatric recipients. Key strengths include the short fWIT achieved, which may contribute to successful graft function, and a DGF rate lower than previously reported. In conclusion, DCD kidney allografts may represent a feasible option for pediatric recipients when used under carefully selected conditions.

## Supplementary Information

Below is the link to the electronic supplementary material.

ESM 1 Supplementary Material 1 (DOCX 248 KB)

Graphical abstract (PPTX 126 KB)

## Data Availability

The data analyzed in the current study are available from the corresponding author upon reasonable request.

## Abbreviations

CIT: Cold Ischemia Time
DBD: Donation after Brain Death
DCD: Donation after Circulatory Death
DGF: Delayed Graft Function
eGFR: Estimated Glomerular Filtration Rate
HLA: Human Leukocyte Antigen
ONT: National Transplant Organization
NT: Normothermic
RR: Rapid Recovery
REPIR I: Spanish Pediatric Registry of Renal Replacement Treatment
KT: Kidney Transplant
WIT: Warm Ischemia Time
tWIT: Total Warm Ischemia Time
fWIT: Functional Warm Ischemia Time
CPR: Cardiopulmonary Resuscitation

## Declarations

### Data transparency

All authors ensure that all data and materials support the published claims and conform to the standards of the field.
