# Supplementary material for: Pediatric kidney transplantation using donors after circulatory death: a national experience from Spain
Source: Pediatr Nephrol. 2026 Apr 14;41(9):3047–58. doi: 10.1007/s00467-026-07235-4 (PMC13424223; doi:10.1007/s00467-026-07235-4)
Supplement: Supplementary file 1 — Supplementary Material 1 (DOCX 248 KB) [file 467_2026_7235_MOESM1_ESM.docx]

**Pediatric kidney transplantation using donors after circulatory death: a national experience from Spain**

- María Herrero-Goñi, MD. Department of Pediatric Nephrology. IIS BioBizkaia Health Research Institute. Cruces University Hospital. University of Deusto. University of the Basque Country UPV/EHU. Barakaldo, Bizkaia, Spain.
  - Department of Pediatric Nephrology. Cruces University Hospital. Cruces place. 48903, Barakaldo, Bizkaia, Spain.
  - Corresponding author: [mherrero901@ikasle.ehu.eus](mailto:mherrero901@ikasle.ehu.eus)
  - ORCID: 0000-0001-6501-0830
- Mireia Aguirre Meñica, MD. Department of Pediatric Nephrology. IIS BioBizkaia Health Research Institute. Cruces University Hospital. Barakaldo, Bizkaia, Spain.
  - ORCID: 0009-0009-8955-087X
- Alejandro Zarauza Santoveña, MD. Department of Pediatric Nephrology. La Paz University Hospital. Madrid, Spain.
  - ORCID: 0000-0002-9415-8911
- Victor Perez-Beltran, MD. Department of Pediatric Nephrology. University Hospital Vall d'Hebron. Barcelona, Spain.
  - ORCID: 0000-0002-9297-371X
- Yolanda Calzada, MD. Department of Pediatric Nephrology. Sant Joan de Déu Hospital. Barcelona, Spain.
  - ORCID: 0000-0002-0635-4200
- David Canalejo González, MD. Department of Pediatric Nephrology. Virgen del Rocío University Hospital. Sevilla, Spain.
  - ORCID 0009-0008-2301-8445.
- Ana Adell Sales, MD. Department of Pediatric Nephrology. La Fe University and Polytechnic Hospital. Valencia, Spain.
- Olalla Alvarez Blanco, MD. Department of Pediatric Nephrology. Gregorio Marañón Hospital. Madrid, Spain.
  - ORCID: 0000-0003-2703-6559
- Iñaki Bilbao-Villasante, MD. Department of Anesthesiology and Intensive Care. Department of Transplantation. Regional Transplant Coordination. Cruces University Hospital. Barakaldo, Bizkaia, Spain.
  - ORCID: 0009-0002-6944-4622
- Spanish Pediatric Registry of Renal Replacement Treatment (REPIR I).

**SUPPLEMENTAL DIGITAL CONTENT**

**Online Resource 1**

Royal Decree 1723/2012, of 28 December, regulates the procurement, clinical use, and territorial coordination of human organs intended for transplantation and establishes quality and safety requirements.

Diagnosis of Death Based on Circulatory and Respiratory Criteria:

1. Diagnosis:

a) Diagnosis of death based on circulatory and respiratory criteria requires unequivocal confirmation of the absence of circulation and spontaneous breathing for at least 5 minutes.

b) As a prerequisite for diagnosis and certification of death based on circulatory and respiratory criteria, one of the following conditions must be verified:

-Advanced cardiopulmonary resuscitation (CPR) maneuvers have been performed for an adequate period without success. This period and maneuvers should be adjusted based on the patient's age and the circumstances of the circulatory-respiratory arrest. Advanced CPR protocols periodically published by relevant scientific societies must be followed at all times. In cases of body temperature ≤32°C, the body must be rewarmed before establishing the irreversibility of circulatory-respiratory failure and, therefore, the diagnosis of death.

-CPR is deemed inappropriate on medical and ethical grounds, in accordance with recommendations from relevant scientific societies.

c) Absence of circulation must be demonstrated by at least one of the following:

-Asystole on continuous electrocardiography.

-Absence of blood flow on invasive arterial blood pressure monitoring.

-Absence of aortic flow on echocardiography.

If scientific and technical advances permit, other instrumental tests providing absolute diagnostic certainty may be used.

2. Maneuvers for Viability Maintenance and Organ Preservation:

a) Preservation procedures may be initiated only after the medical team responsible for the patient has documented death in writing, specifying the time of death.

b) When judicial authorization is required (per Article 9.5 of this Royal Decree), the following applies:

-In cases referenced in 1.b).1), measures to maintain organ perfusion may be resumed, and the investigating court notified of a potential donor. Preservation may begin after a favorable court response or 15 minutes without court-imposed restrictions. Prior to these procedures, collect a 20-mL blood sample and, if possible, 20 mL urine and 20 mL gastric juice (per chain-of-custody protocol), to be provided to the court along with any additional samples or data requested. Preservation procedures may then commence.

-In cases referenced in 1.b).2), notify the investigating court of the potential donor and case circumstances before starting procedures; proceed per court guidelines.

-In both prior cases, organ procurement may occur only after obtaining corresponding judicial authorization (per Article 9.5).

**Figure 1 Management protocol for a potential organ donor following the withdrawal of life-sustaining treatment decision.**

**
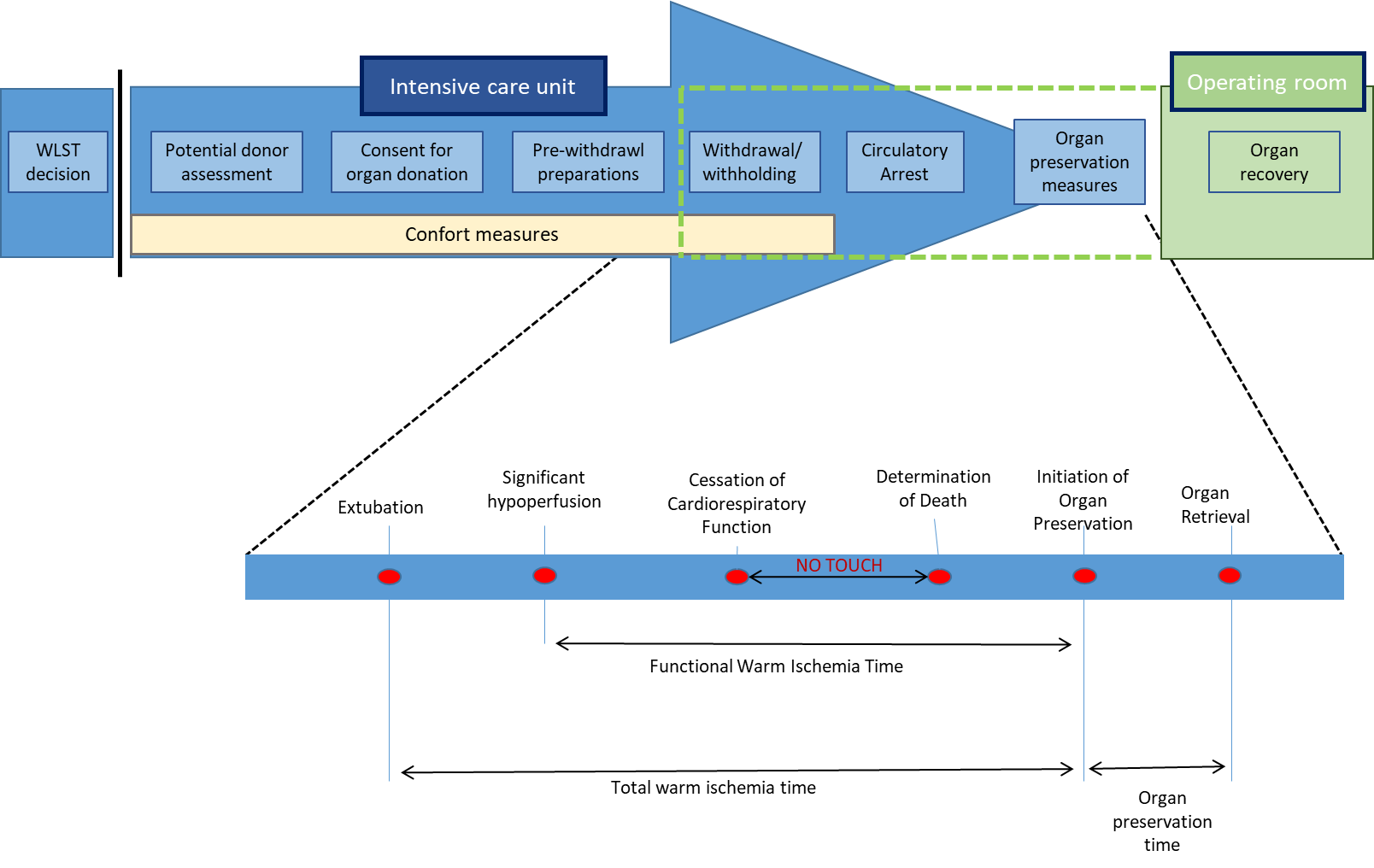
**

WLST, Withdrawal of life-sustaining treatment

**Online Resource 2**

**Table 1a Immunosuppressive regimen in 64 KT recipients from 58 DCD donors**

| Recipients´ characteristics (n=64) | Mean (± SD),  Median [IQR: 25^th^, 75^th^] or number (%) |
| --- | --- |
| Induction immunosuppression | |
| Basiliximab | 50 (72.1%) |
| Anti-thymocyte globulins | 14 (21.9%) |
| Calcineurin inhibitor + antimetabolite + corticosteroids | 62 (96.8%) |
| Others | |
| Azathioprine or everolimus | 2 (3.1%) |
| Plasmapheresis/immunoadsorption+ Immunoglobulins+ Rituximab | 3 (4.6%) |
| Plasmapheresis/immunoadsorption | 1 (1.56%) |
| Rituximab | 1 (1.56%) |
| Eculizumab | 1 (1.56%) |

DCD, donation after circulatory death; IgG, immunoglobulin G.

**Table 1b Comparison of recipient characteristics between patients who received a preemptive KT and those on dialysis**

|  | Preemptive transplant (n=13) | Chronic dialysis (n=51) | P value |
| --- | --- | --- | --- |
| Time on the waiting list (months) | 1.5 [0.9;4.4] | 3.40 [1;8.75] | *n.s.* |
| HLA mismatches |  | | |
| A mismatches (0,1,2) | 0,5,8 | 3,24,24 | *n.s.* |
| B mismatches (0,1,2) | 0,2,11 | 0,18,33 |  |
| DR mismatches (0,1,2) | 3,7,3 | 9,24,18 |  |

HLA, human leukocyte antigen; N.S., non-significant p-value

**Online Resource 3**

**Figure 2**

**Figure 2a Cold ischemia time *vs.* Delayed Graft Function**

**
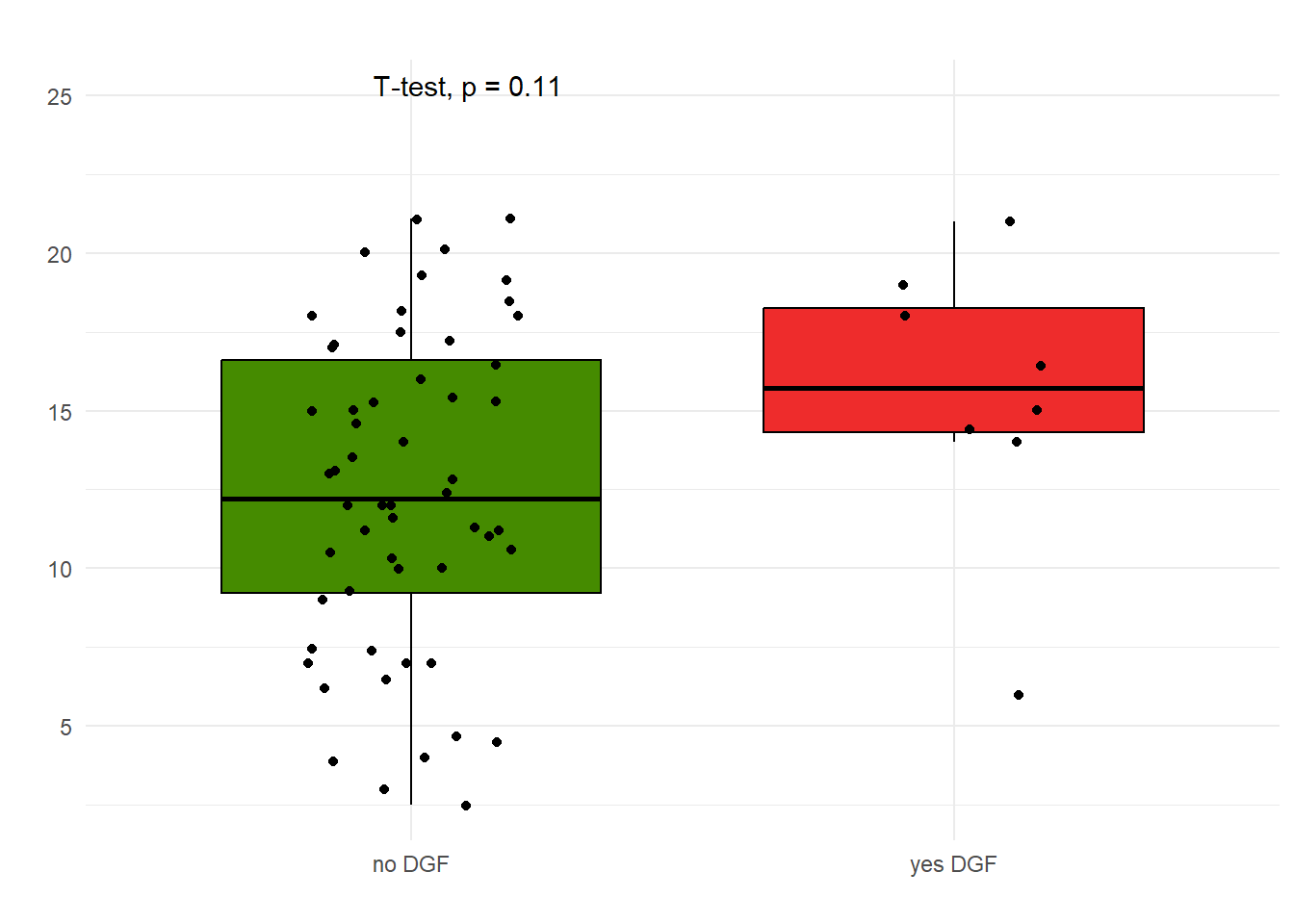
**

DGF, delayed graft function

**Figure 2b Functional warm ischemia time *vs.* Delayed Graft Function
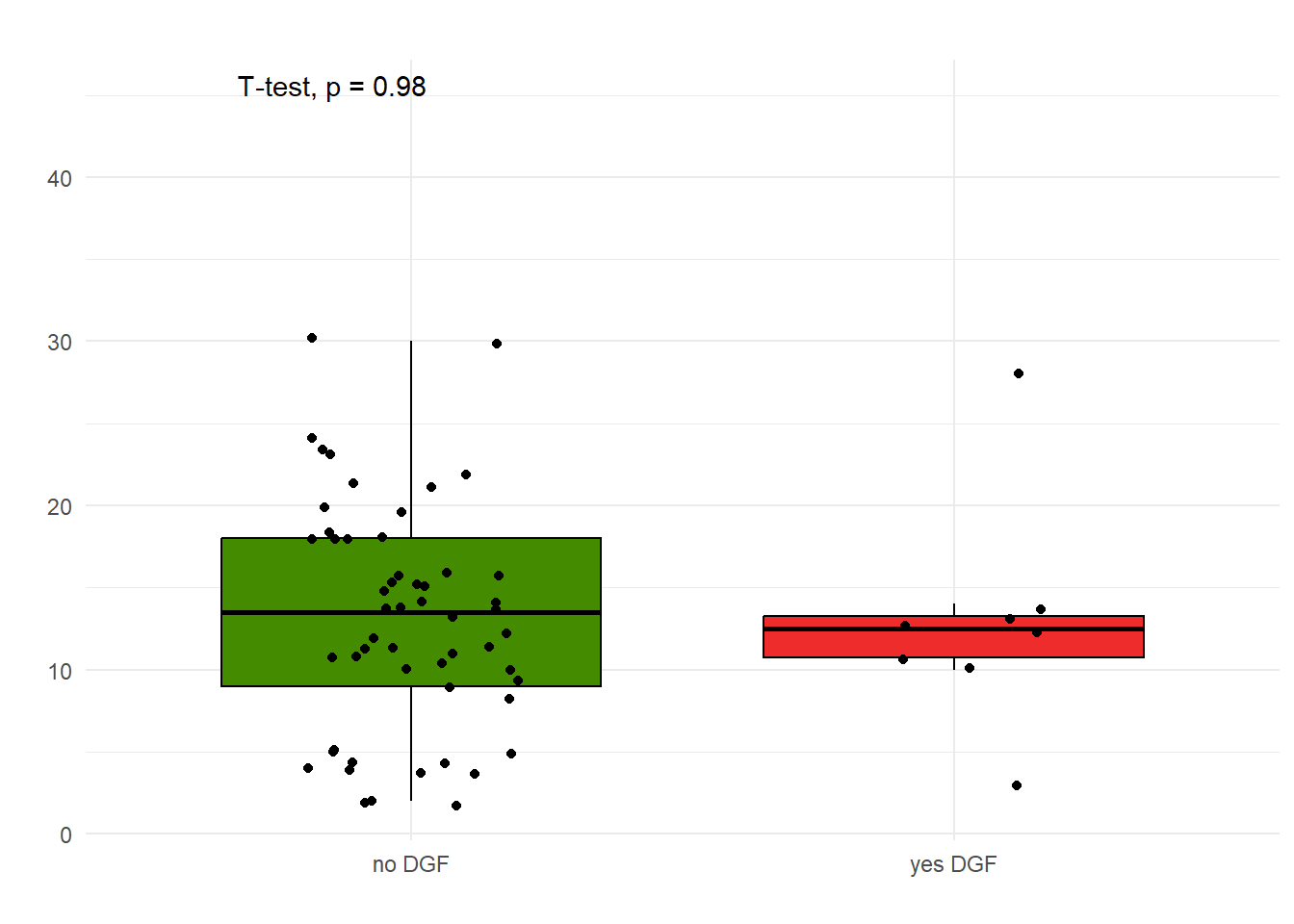
**

DGF, delayed graft function

**Figure 2c Relation between Delayed Graft Function and estimated Glomerular Filtration Rate one month after transplantation**

**
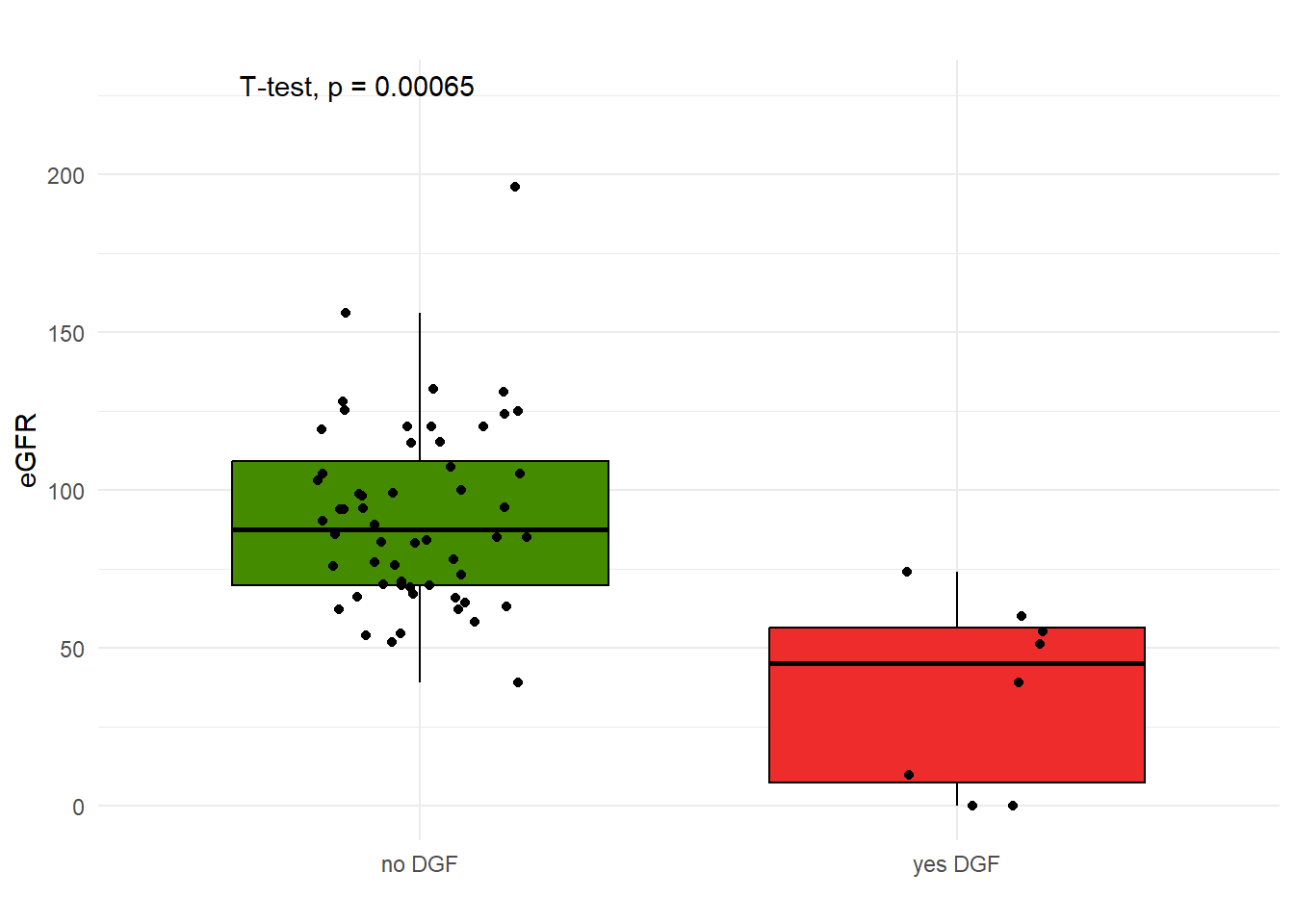
**

eGFR, estimated glomerular filtration rate; DGF, delayed graft function

**Figure 2d Relation between Delayed Graft Function and estimated Glomerular Filtration Rate one year after transplantation**

**
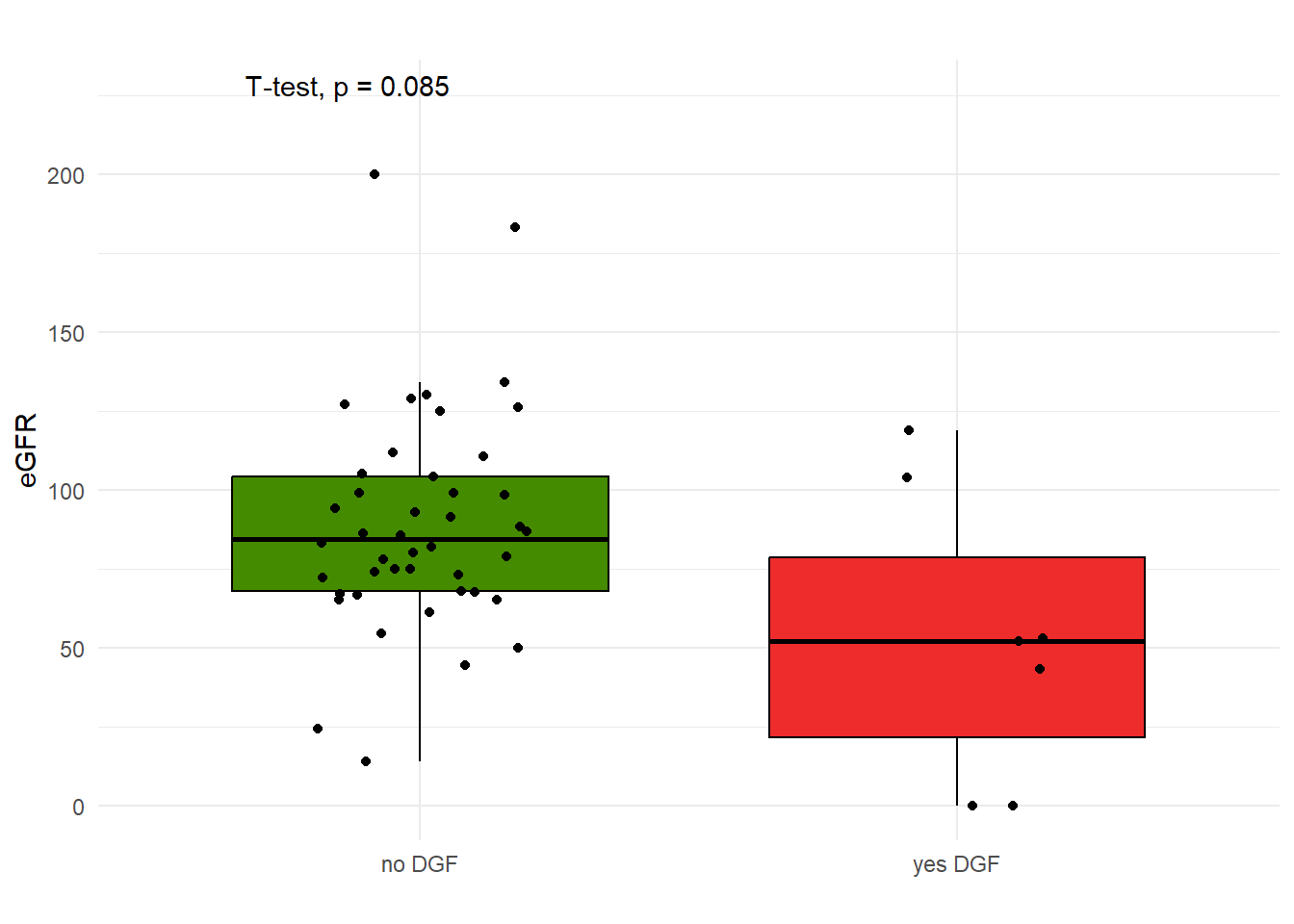
**

eGFR, estimated glomerular filtration rate; DGF, delayed graft function

**Online Resource 4**

**Figure 3**

**Figure 3a: Cold ischemia time and estimated Glomerular Filtration Rate one month after transplantation**

**
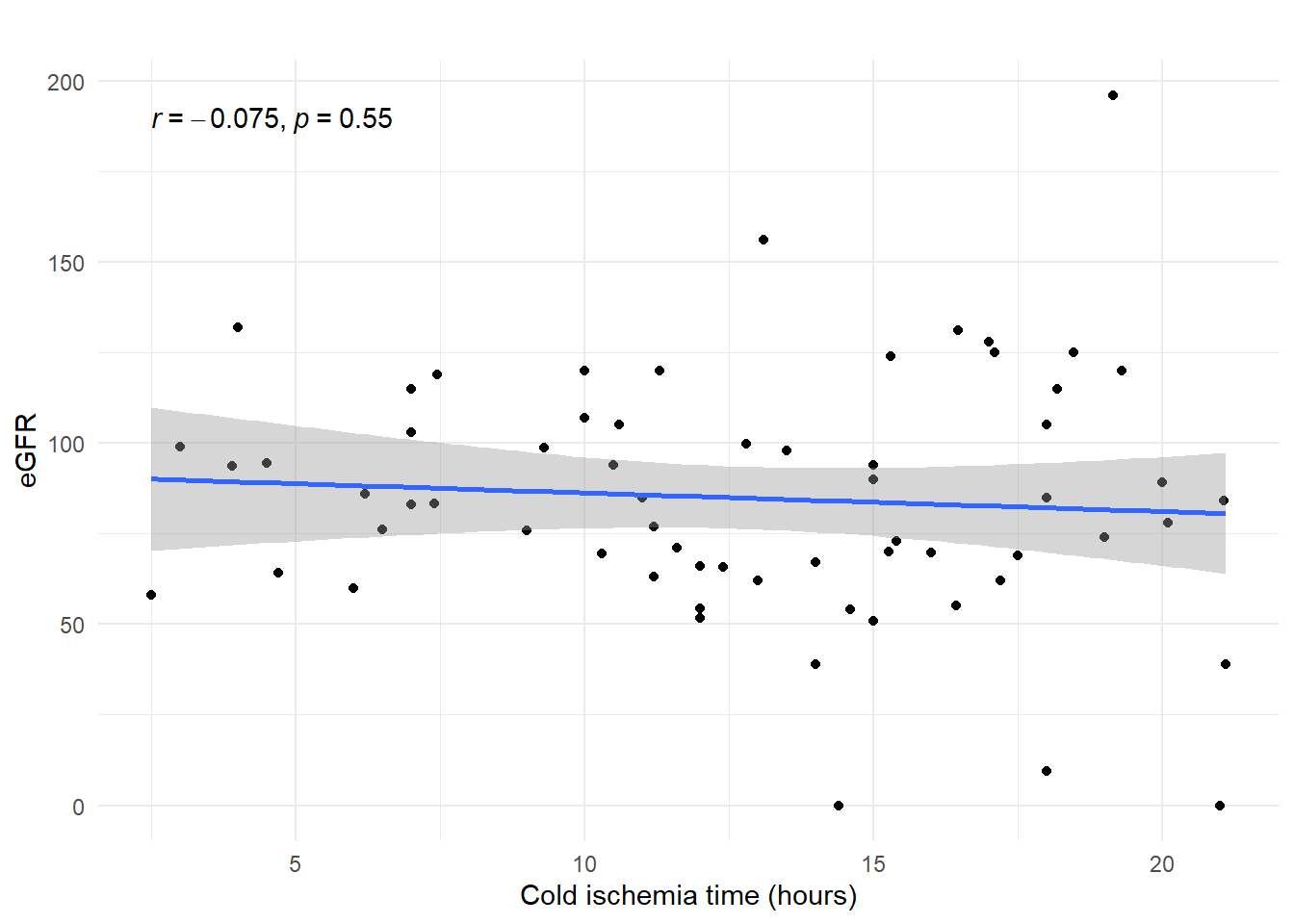
**

eGFR, estimated glomerular filtration rate

**Figure 3b: Cold ischemia time and estimated Glomerular Filtration Rate one year after transplantation**

**
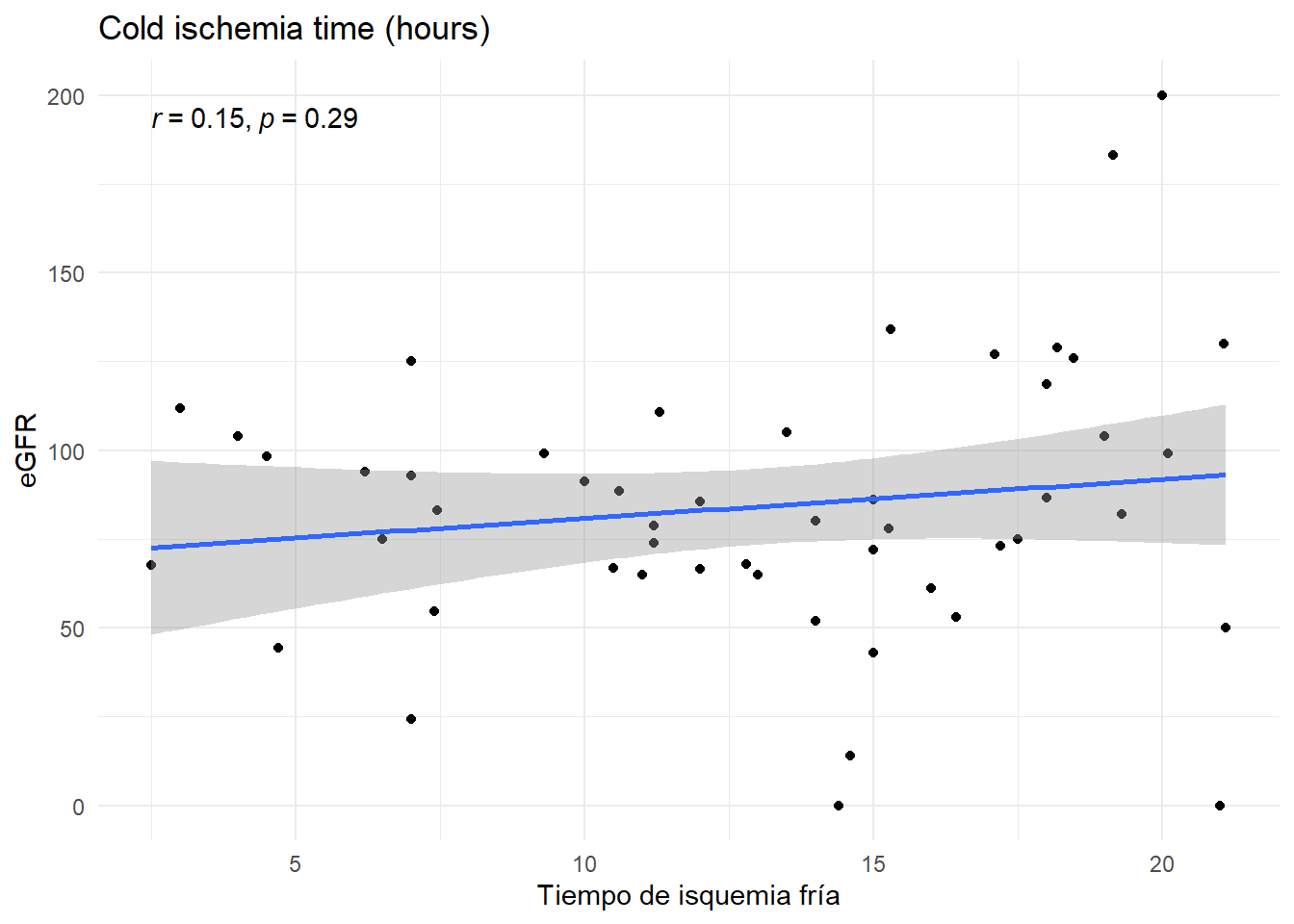
**

eGFR, estimated glomerular filtration rate

**Figure 3c Functional warm ischemia and estimated Glomerular Filtration Rate one month after transplantation**

**
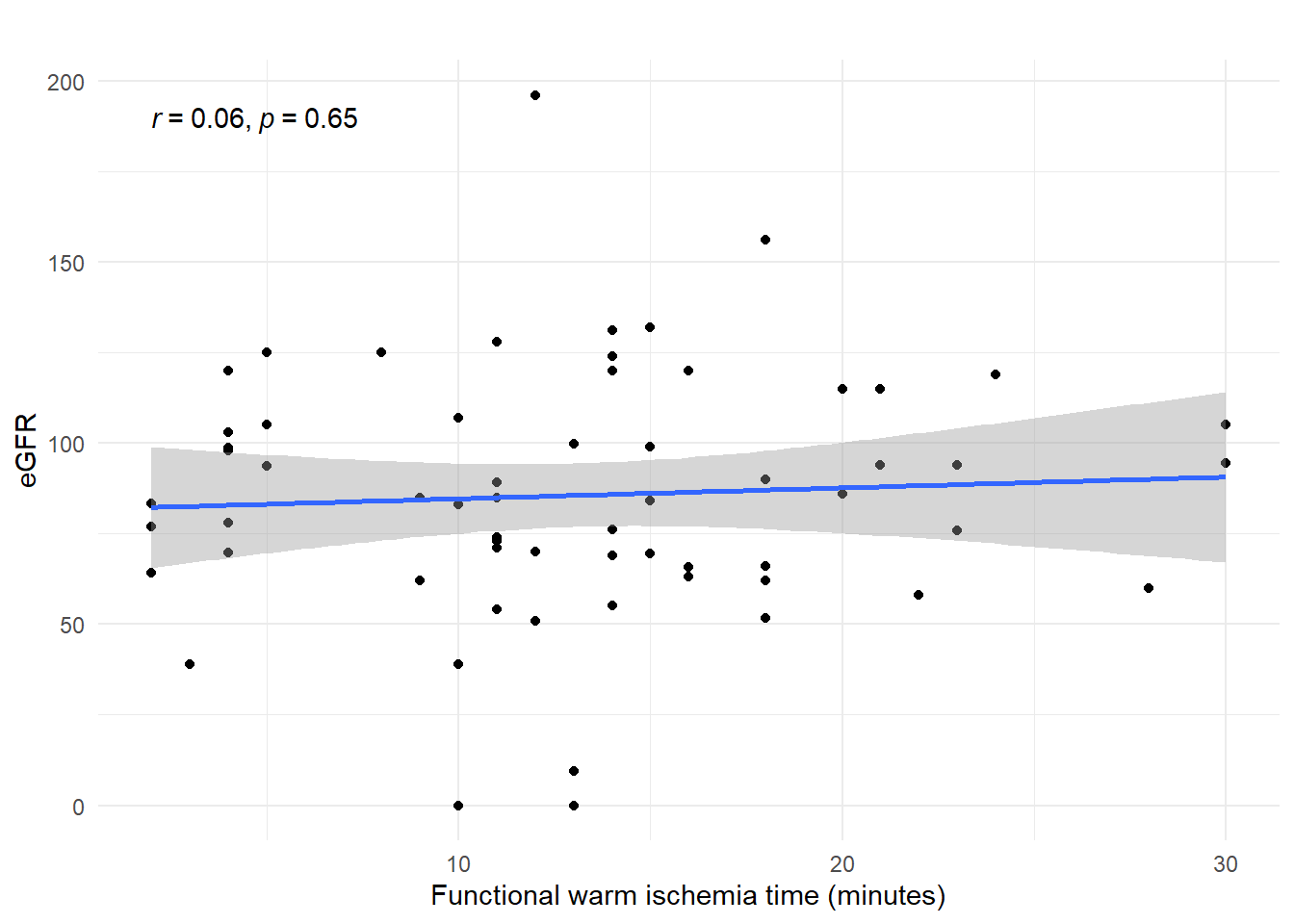
**

**Figure 3d Functional warm ischemia and estimated Glomerular Filtration Rate one year after transplantation**

**
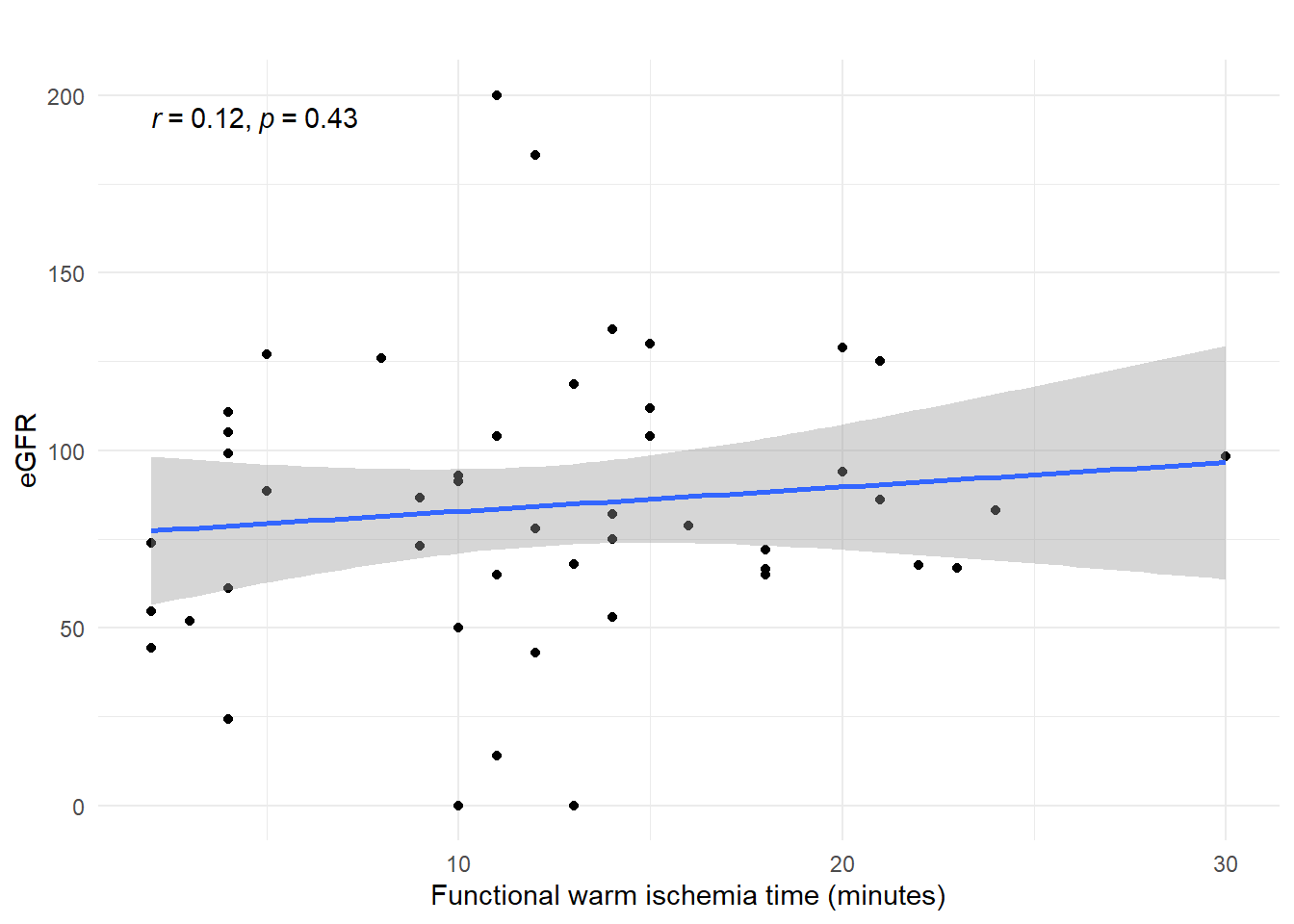
**
