# Supplementary material for: Pediatric kidney transplantation using donors after circulatory death: a national experience from Spain
Source: Pediatr Nephrol. 2026 Apr 14;41(9):3047–58. doi: 10.1007/s00467-026-07235-4 (PMC13424223; doi:10.1007/s00467-026-07235-4)
Supplement: Supplementary file 2 — Graphical abstract (PPTX 126 KB) [file 467_2026_7235_MOESM2_ESM.pptx]

## Slide 1
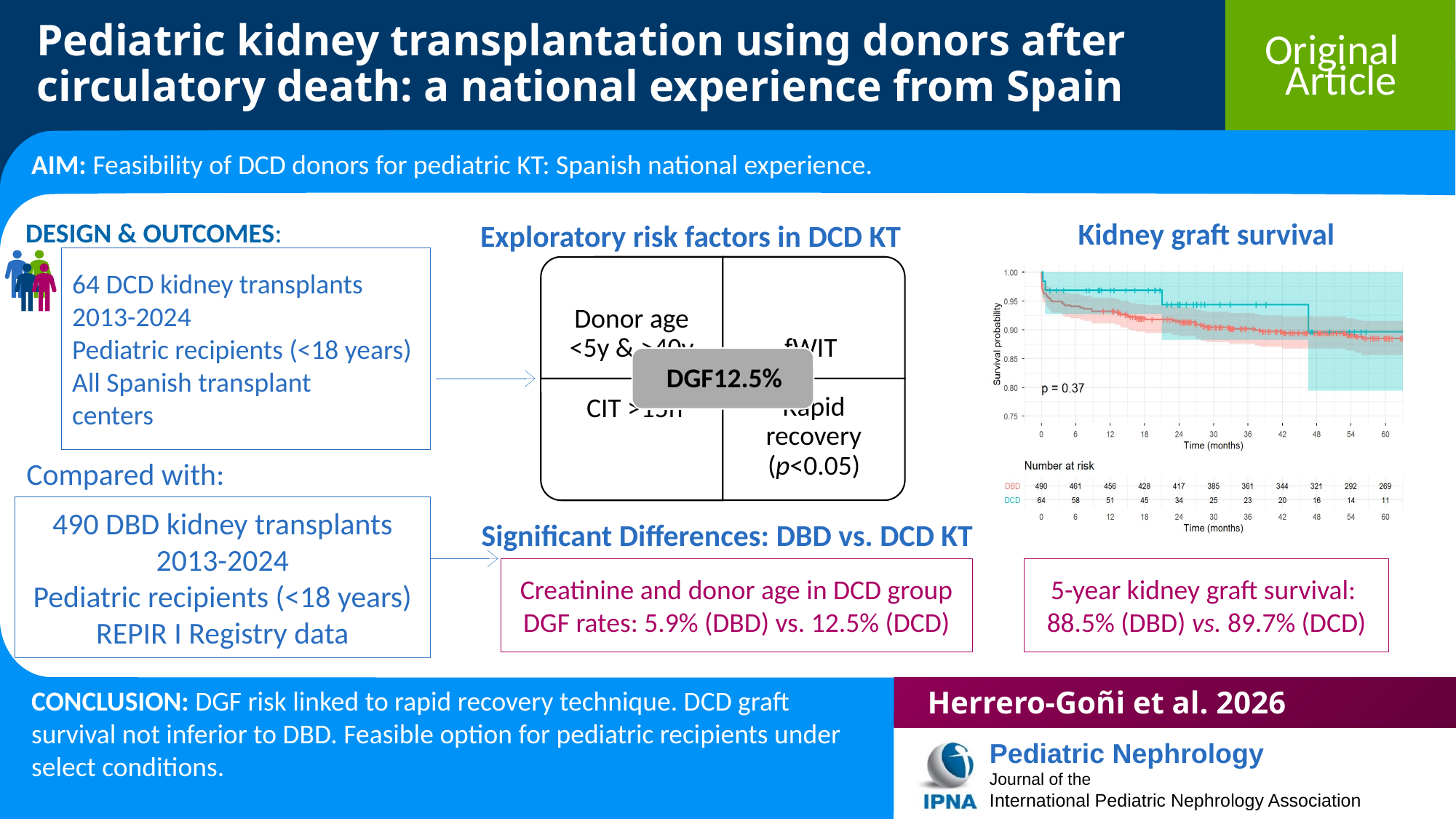

Pediatric kidney transplantation using donors after circulatory death: a national experience from Spain
AIM: Feasibility of DCD donors for pediatric KT: Spanish national experience.
Kidney graft survival
DESIGN & OUTCOMES:
Exploratory risk factors in DCD KT
64 DCD kidney transplants
2013-2024
Pediatric recipients (<18 years)
All Spanish transplant
centers
Compared with:
490 DBD kidney transplants
2013-2024
Pediatric recipients (<18 years)
REPIR I Registry data
Significant Differences: DBD vs. DCD KT
Creatinine and donor age in DCD group
DGF rates: 5.9% (DBD) vs. 12.5% (DCD)
5-year kidney graft survival:
88.5% (DBD) vs. 89.7% (DCD)
CONCLUSION: DGF risk linked to rapid recovery technique. DCD graft survival not inferior to DBD. Feasible option for pediatric recipients under select conditions.
Herrero-Goñi et al. 2026
